# Approach to electrochemical modulating differential extended X-ray absorption fine structure

**DOI:** 10.1107/S1600577522005616

**Published:** 2022-06-08

**Authors:** Wenjie Xu, Guikai Zhang, Hongwei Shou, Jia Zhou, Shuangming Chen, Shengqi Chu, Jing Zhang, Li Song

**Affiliations:** a National Synchrotron Radiation Laboratory, CAS Center for Excellence in Nanoscience, University of Science and Technology of China, Hefei, Anhui 230026, People’s Republic of China; b Beijing Synchrotron Radiation Facility, Institute of High Energy Physics, Chinese Academy of Science, Beijing 100049, People’s Republic of China; c University of Chinese Academy of Sciences, Beijing 100049, People’s Republic of China; d Department of Materials Sciences and Engineering, School of Chemistry and Materials Sciences, University of Science and Technology of China, Hefei, Anhui 230026, People’s Republic of China; e State Key Laboratory of Particle Detection and Electronics, Institute of High Energy Physics, Chinese Academy of Science, Beijing 100049, People’s Republic of China

**Keywords:** differential XAS, modulation excitation spectrum, phase-sensitive detection, electrocatalysis

## Abstract

The differential XAFS technique holds promise for detecting surface changes, which benefits many chemical applications. Phase-sensitive detection (PSD) analysis based on modulated excitation spectroscopy experiments is expected to obtain a high-quality difference spectrum, while the mathematical relationship and experiment parameters remain to be discussed. In this article, an approach to obtaining the difference spectrum from the PSD demodulated spectrum is described and its applicability in different experiment settings is discussed. The results indicate that the demodulated spectrum is almost equal to the difference spectrum when the modulating period is 20 times larger than the relaxation time constant. This approach was subsequently applied to an electrochemical modulation experiment and the demodulated spectrum was analyzed. A reversible lattice shrinking is observed via the fitting of demodulated spectra, which is proportional to the charge amount on the electrode. This approach could be used to quantitatively analyze the modulated excitation XAS data and holds promise for a wide range of electrochemical studies.

## Abstract 2

The quantitative relationship between phase-sensitive detection demodulation and difference spectrum is derived and used to quantitatively analyze the electrochemical modulation excitation X-ray absorption spectrum.

## 1. Introduction

Electrocatalysis has attracted tremendous attention due to its extensive application in the chemical industry as well as its promise for developing renewable energy sources. As a basic reaction in many electrolysis processes, the oxygen evolution reaction (OER) commonly occurs if no other sacrificial ions exist, but is usually sluggish as a four-electron process, especially in an acid environment (An *et al.*, 2021 ▸). Efficient catalysts are needed to reduce the energy barrier, while the rational design of the catalysts still meets obstacles regarding the insufficient fundamental understanding of the catalysts’ atomic structure and the adsorption–desorption process (Jiang *et al.*, 2018 ▸). Recent development of the *in situ* technique made it possible to discover the real state of the catalyst during the catalytic process and many instances of the structural response towards potential have been proved (Ding *et al.*, 2021 ▸). It is vital to continuously develop characterization methods for electrocatalysis research.

For revealing the structural evolution of catalysts, *in situ* X-ray absorption fine structure (XAFS) has been applied frequently (Cao, Liu *et al.*, 2021 ▸). However, XAFS comes from an average of all the atoms of the detected elements, which makes the spectral change induced by surface processes not so obvious. To strengthen only the change of different states, making use of the difference spectrum (diffXAS, Δμ) is a powerful analysis technique. Generally speaking, diffXAS is the difference in the spectra obtained in different states,

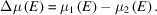

Its mathematical form is well defined, thus allowing quantitative analysis of the change in the system. In a representative work, Pettifer *et al.* (2005 ▸) enhanced the accuracy of the interatomic distance into femtometres using diffXAS. In the X-ray absorption near-edge structure (XANES) part, Δμ-XANES is used to compare the oxidation state and adsorption species (Ramaker & Koningsberger, 2010 ▸). However, it is difficult to obtain a high-quality difference spectrum in the extended X-ray absorption fine-structure (EXAFS) range with static measurement, especially for *in situ* research where the solution environment contributes to a low signal-to-noise (S/N) ratio. The modulated excitation spectrum (MES) has been used to detect the tiny difference between different states given by periodic circumstances or fields which leads to a periodical response of the measured system (Ferri *et al.*, 2011 ▸). For extraction of the periodical signal with known frequency, there is a mature solution in electronics called phase-sensitive detection (PSD). PSD is a narrow-band technique that can filter noise, thus enhancing the signal-to-noise ratio. Our group has used a PSD-based electronic device to obtain difference XAFS via pressure modulation (Chu *et al.*, 2012 ▸). One can also collect MES data via time-resolved methods and perform PSD analysis offline (König *et al.*, 2012 ▸). However, the response of the system to external conditions may not be so rapid and show kinetic behavior. If the stimulation period is in the range of the relaxation time of the system, amplitude decay and phase lag of the response will occur, which depend on the modulation frequency (Baurecht & Fringeli, 2001 ▸; Müller & Hermans, 2017 ▸). In this regard, it is incorrect to use the amplitude obtained from the PSD analysis as the difference spectrum. Instead, the phase-sensitive properties of PSD can be used to analyze the dynamic properties of the system (Chiarello & Ferri, 2015 ▸). Still, PSD applied to MES data results in a special kind of difference spectrum between excited and non-excited states (Gremlich & Yan, 2000 ▸), thus allowing qualitative analysis. In electrocatalysis research, MES experiments and PSD analysis have been used to catch the dynamic changes in adsorption species and surface structure (Czioska *et al.*, 2021 ▸; Lawley *et al.*, 2022 ▸; Ebner *et al.*, 2022 ▸), which proved the great potential of this method. However, if the quantitative relationship between PSD demodulated XAS and the diffXAS could be elucidated, PSD analysis can give more quantitative results. In fact, amplitude attenuation and phase lag are related to the modulation period and relaxation time constant of the measured system as discussed in former modulation excitation infrared spectrum research (Baurecht & Fringeli, 2001 ▸; Urakawa *et al.*, 2006 ▸), which could also be used in the ME-XAS experiment.

In this article, we obtained the mathematical relationship between the difference spectrum and PSD demodulated ME-XAS data in a slow-relaxation system using an electrochemical system as an example. Our results show that if the modulation period is 20 times more than the system relaxation time constant, after a certain correction, the quantitative difference between the PSD demodulated spectrum and the difference spectrum can be less than 2% while the S/N ratio could be enhanced using PSD analysis. Thus, it is possible to obtain the difference spectrum from the PSD method. This method was further applied to the electrochemical system and a reversible shrinking of bond length along with increasing potential was found. This study provides a feasible approach for quantitative analysis of ME-XAS data, which may inspire many other modulated excitation experiments.

## 2. Theory

### 2.1. Fourier series

According to the well known Fourier decomposition, any periodic function can be written as a superposition of a series of sinusoidal functions in a finite interval. Let its period be *T*; in the interval [0, *T*], it can be written as the following Fourier series expansion,

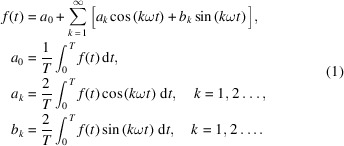

The coefficients *a*_*k*_ and *b*_*k*_ can be seen as the projection of a vector on the *x*-axis and the *y*-axis, respectively. Therefore, the Fourier series can be further written in the following form,

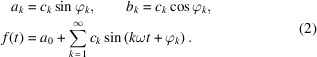

Thus, a periodical signal can be written as a superposition of a series of sine functions with different amplitudes (modulus) and phase angles, which forms the basis of frequency domain analysis.

### 2.2. Modulation excitation spectrum and phase-sensitive detection

If a periodically changing external circumstance or field (sine wave, square wave, *etc*.) is applied to a system during a spectrum test, the resulting spectrum signal will also oscillate at the same frequency. In an ME-XAS experiment, the absorbance signal μ(*E*, *t*) can be expressed in the form of a Fourier series,

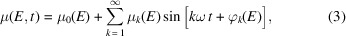

where *k* is the order of the Fourier transform, ω is the frequency of the external excitation, and μ_*k*_ and φ_*k*_ are the corresponding amplitude and phase angle. To extract the amplitude and phase information, the basic formula for obtaining the Fourier transform coefficients can be used,

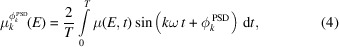

where 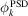 is the PSD demodulating phase angle. This is the basic function of PSD. If 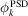 = 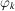 , then 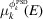 = 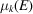 , thus the result of this formula is the coefficient of the Fourier series. There are also the following relationships for other values,

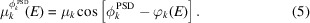

The result of the PSD is therefore phase sensitive. To obtain the phase difference between an input signal (reference signal) with a known phase and an output signal with an unknown phase, one can carry out trigonometric function processing on the results when the demodulation angle equals 0° and 90° via equation (2), or directly find 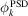 to make 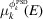 achieve the maximum value.

### 2.3. Phase lag and kinetics

For an ideal system where the output signal has the same phase as the input signal, the system’s response is immediate. However, in a real system, the response has a kinetic behavior which leads to phase lag and amplitude decay. Taking a square-wave input as an example for a more intuitive understanding, the ideal output is as follows,

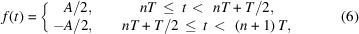

where *T* = 2π/ω is the period and *A*/2 is the amplitude. The result of its Fourier transform is easily obtained,

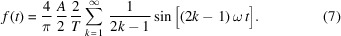

For an ideal square wave, first-order (*k* = 1) sinusoidal demodulation will give the best demodulation angle of 0 and an amplitude of 2*A*/π, which has a clear 2/π multiple relationship with the difference *A*. However, the actual response often exhibits relaxation properties. Consider a common exponential decay relationship which can be described by the following equation, 

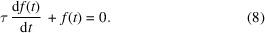

**Figure 1:**
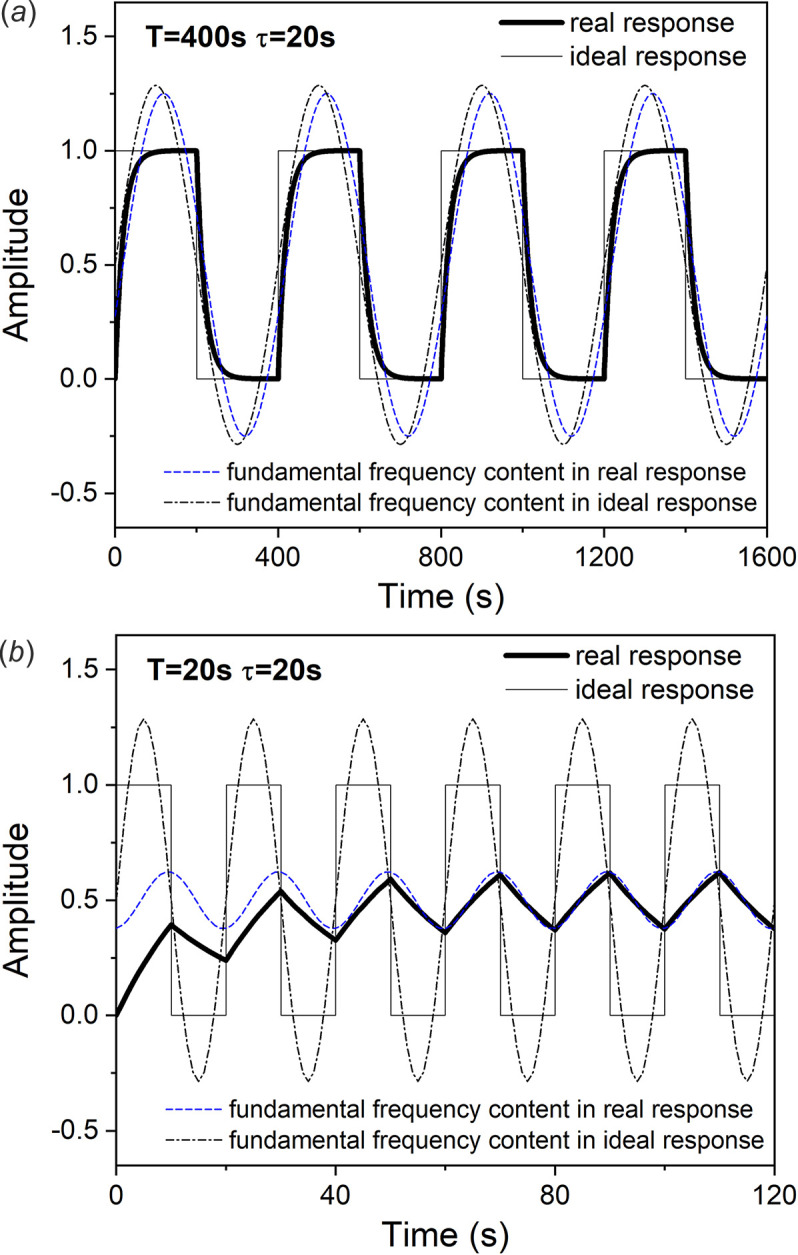
Time response of the first-order system with a square-wave input. The inputting waveform has a different period, 400 s in (*a*) and 20 s in (*b*).

This is called a first-order system. Its step response in the time domain is as follows,

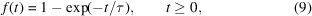

where τ is usually called the time constant and describes how fast the system changes. Inputting a square wave with different periods will result in the output shown in Fig. 1 ▸. It can be seen that when the modulation period is much larger than the time constant, the relaxation of the system is sufficient. At this time, the fundamental frequency component contained in the signal has a smaller phase delay and amplitude attenuation than a perfect square wave. However, when the modulation period becomes shorter, the oscillation needs several cycles to stabilize first. The amplitude of the waveform is significantly attenuated, and the phase of the fundamental frequency is also significantly delayed. Thus, the phase lag and amplitude attenuation depend on the modulation period and relaxation time constant. Using this, we can obtain the phase lag through PSD analysis and analyze the kinetics of the system.

### 2.4. Transfer function

If the response of the system is linear, the above-mentioned frequency attenuation and phase lag can be analyzed using the transfer function. In the Laplace domain, the transfer function *G*(*s*) relates the input function *U*(*s*) of the system to the output function *Y*(*s*),

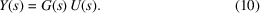

When the input function is a sinusoidal signal with frequency ω, the output signal can be expressed as

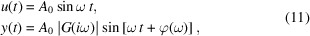

where |*G*(*i*ω)| is the modulus of *G*(*i*ω) in the complex plane, and φ(ω) is the phase angle, so the amplitude attenuation and phase delay between the sinusoidal input and output of different frequencies can be easily obtained from the transfer function. For the aforementioned exponentially decaying response, the transfer function has the following form,

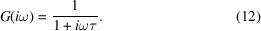

The amplitude–frequency characteristics and phase–frequency characteristics can be obtained,

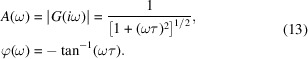

**Figure 2:**
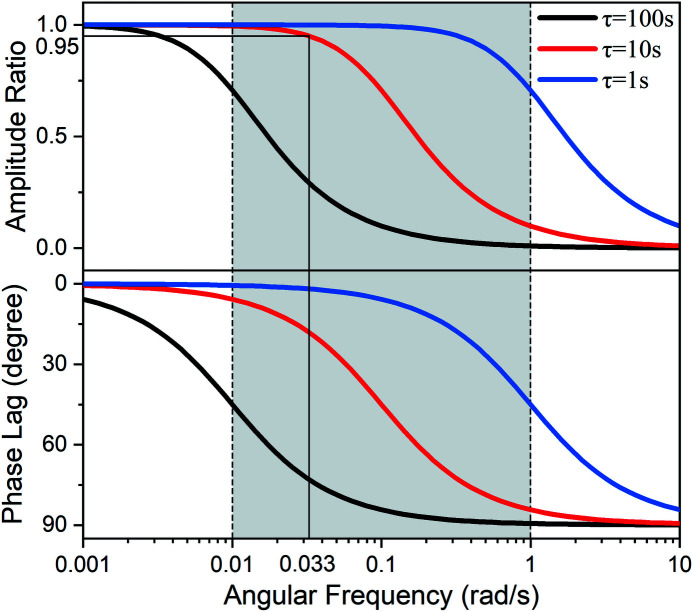
Bode plot of the first-order system.

A plot of these two relationships is called a Bode plot. It can be seen that the amplitude attenuation and frequency change are only related to ωτ, as shown in Fig. 2 ▸.

When the frequency is fixed, increasing the time constant also leads to an increase in the phase delay, so the kinetics of the system can be analyzed by obtaining the phase lag. For different systems, if the transfer function can be obtained according to its physical model, the phase lag can be used to obtain the kinetic parameters. When performing dynamic analysis, it is best to select the region where the phase delay changes rapidly with frequency, generally set to 0.1 < ωτ < 10, which corresponds to the region in the gray background of the τ = 10 curve in Fig. 2 ▸.

### 2.5. Relationship between PSD and difference spectrum

According to the above analysis, the relationship between PSD amplitude and absolute amplitude can be obtained when the transfer function is known. In the actual MES experiment, the frequency of the modulating external field is known, and the phase difference of the response signal can be given by the PSD so that the dynamic parameter τ can be obtained, and the ratio of the amplitude attenuation at a specific frequency can be obtained from τ, thereby obtaining the absolute amplitude. The absolute amplitude has a definite relationship with the difference value since the input waveform is known. This is the mathematical relationship between the PSD result and the difference spectrum.

When a sine wave is input, the output of a linear system is also a sine wave. For a square-wave input, it can be decomposed as a superposition of infinite sine waves after the Fourier transform. Due to the additivity of the linear system, it can be considered that the sine waves of each frequency component contained in the square wave will output the corresponding sine wave of the same frequency but with different amplitude and phase according to the relationship in the Bode diagram. The superposition of these signals becomes the output waveform. According to the aforementioned amplitude correction method, the following formula can be obtained to describe how to use the PSD analysis of the fundamental frequency to obtain the difference spectrum,

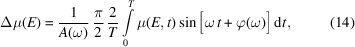

where π/2 is the coefficient between the amplitude of the square-wave signal and the fundamental frequency sine signal. φ(ω) can be obtained by finding the demodulation angle used to maximize the integral in PSD analysis. Then, the amplitude attenuation *A*(ω) is obtained from the kinetic parameters through equation (13). There is also a benefit to using square-wave modulation that the kinetic parameters can be obtained from analyzing the relaxation curve. Although the absolute amplitude can be obtained theoretically, the model is usually simplified and the acquisition of the kinetic parameters may be inaccurate, so there is also an error in the acquisition of the absolute amplitude, which will also lead to inaccurate corrections. Therefore, to obtain the differential spectrum from the PSD, amplitude attenuation should be as small as possible. We recommend controlling the experimental conditions so that the correction value is less than 5%, which needs ωτ < 0.33, or approx,imately, *T*/τ > 20, as seen in Fig. 2 ▸. For higher accuracy, the value of ωτ should be smaller, as in experiments with fast response such as high pressure (Chu *et al.*, 2012 ▸), magnetostriction (Ruffoni *et al.*, 2008 ▸), *etc*.

Finally, it should be emphasized that in a nonlinear system the input sinusoidal signal will output higher harmonics. So there is no one-on-one correspondence in the frequency domain, and there is no transfer function, making kinetics analysis very difficult. Still, if the response of the system is close enough to a square wave by setting the experimental conditions, the differential spectrum can be obtained within an acceptable error range.

## 3. Experimental

### 3.1. IrO_*x*_ electrode preparation and electrochemical measurement

The IrO_*x*_ electrode used for modulating differential XAFS experiment is synthesized by calcination of Ir(III) acetate (Shanghai Macklin Biochemical Co. Ltd, Ir 50–54%). The Ir(III) acetate was dissolved in DI water to form a 60 g L^−1^ homogeneous solution. Then the solution was dropped onto carbon paper (Toray, TGP-H-060) at 200 µL cm^−2^ and dried at room temperature. Finally, the carbon paper was annealed in air for 2 h at 250°C. A similar process was used to prepare the electrode for electrochemical cyclic voltammetry (CV) test by 100-fold diluent loading.

All electrochemical measurements as well as the *in situ* test were performed using a CHI760e (CH Instruments, China) electrochemical workstation in a standard three-electrode setup with graphite rods as a counter electrode and an Hg/Hg_2_SO_4_ electrode as a reference electrode. The electrolyte was 0.5 *M* H_2_SO_4_ with Ar saturation, and all potentials were calibrated to the reversible hydrogen electrode (*E*_RHE_ = *E*_Hg/Hg2SO4_ + 0.652 + 0.059 × pH) accordingly. The scanning rate of the electrochemical CV was 50 mV s^−1^. The period used in the chronoamperometry (CA) test and the following modulation was 400 s.

### 3.2. XAFS measurement and data process

**Figure 3:**
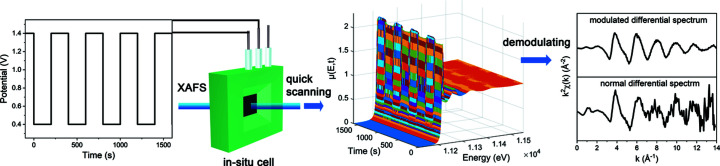
Schematic representation of the testing method of electrochemical modulating differential XAFS.

The experiment setting is shown in Fig. 3 ▸. All the XAFS experiments were performed at the 1W1B beamline at Beijing Synchrotron Radiation Facility (BSRF) (operating at 2.5 GeV with a maximum current of 250 mA). The main optics of this beamline consist of a front slit, vertical collimation mirror, Si(111) fixed-exit double-crystal monochromator, and toroidal mirror. The flux at the sample is of the order of magnitude of 10^11^ photons s^−1^ and the spot size is 0.9 mm (H) × 0.3 mm (V). A homemade electrochemical *in situ* cell is used in transmission mode. The windows on both sides are sealed by Kapton tap with a gap of about 1.5 mm, which is also the thickness of the solution. One end of the carbon paper electrode is connected to the platinum electrode clip (above the liquid surface), and the other end is immersed in the solution. The solution is stagnant as there is almost no gas generation involved in our experiment. The Ir *L*_3_-edge XAFS spectra were collected in QXAFS mode where a full spectrum was acquired in about 5 s. The spectra were continuously recorded with the potential changing for more than four complete periods (about half an hour). By using the *ATHENA* and *ARTEMIS* module of the *IFEFFIT* software package (Ravel & Newville, 2005 ▸), the obtained EXAFS data were processed and fitted. We fitted the IrO_2_ standard sample first to obtain the amplitude attenuation factor 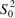 , whose value is 0.77 here. The parameter setting is discussed later.

## 4. Results and discussion

### 4.1. Electrochemical result

**Figure 4:**
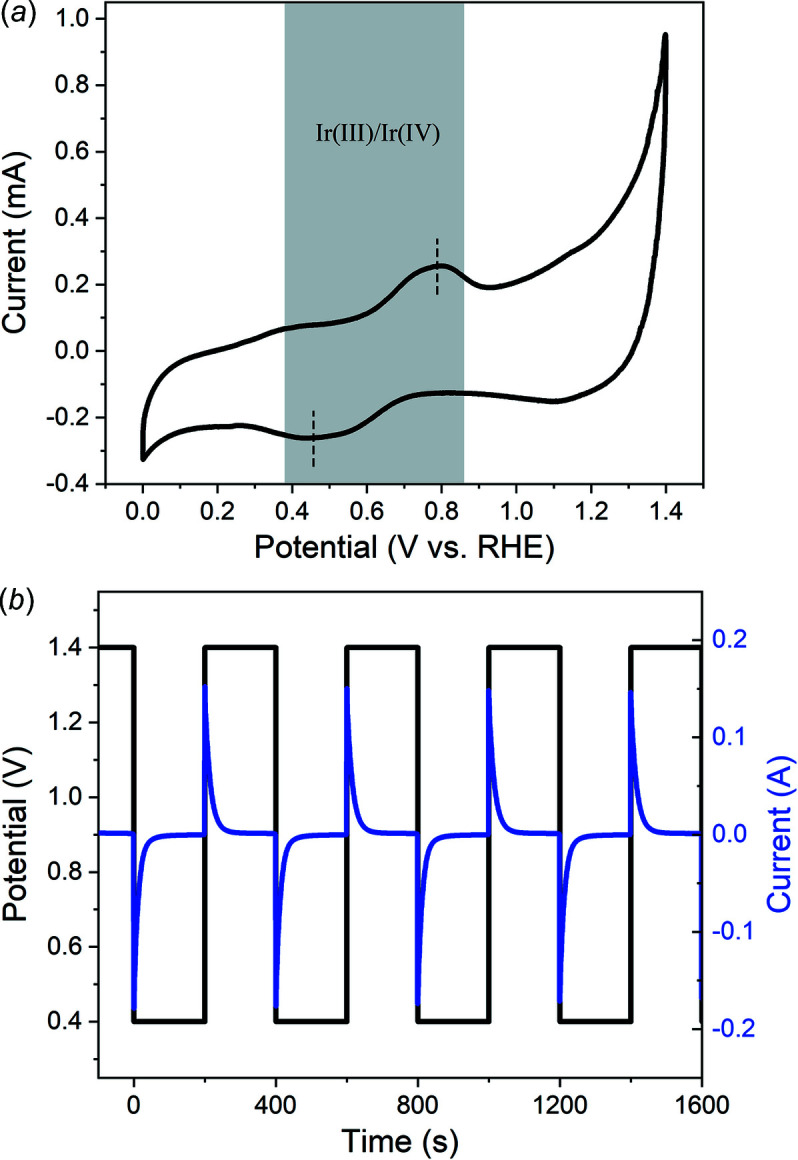
(*a*) CV curve of the as-prepared low-loading IrO_*x*_ electrode. (*b*) CA curve of IrO_*x*_ used in the modulation XAFS test.

Electrochemical CV was performed to illustrate the basic properties of the IrO_*x*_ electrode. As shown in Fig. 4 ▸(*a*), the main oxidation/reduction peaks appear at 0.78 V/0.45 V, which are assigned to the Ir(III)/Ir(IV) redox couple as reported in earlier studies (Nong *et al.*, 2018 ▸; Saveleva *et al.*, 2018 ▸). The CV result determines the potential used for modulation where the low potential should be below 0.45 V and the high potential should be over 0.78 V to make the surface redox reaction occur. Then, we performed CA to reveal the charge–discharge property, where we alternately apply two potentials on the catalyst and record the current as a function of time. Based on the CV result, we choose the low potential at 0.4 V to reduce the high valence surface Ir to the initial state, and the high potential at 1.0 V, 1.2 V and 1.4 V to examine the surface charge property. The result of 0.4 V/1.4 V is shown in Fig. 4 ▸(*b*), where a typical exponential growth and decay of current can be found. The current curve shows a good periodicity, implying the stability of the system. The CA curve of bare carbon paper is also tested (Fig. S1 of the supporting information) and the current is much smaller than for the IrO_*x*_ loaded sample, proving that almost all the capacitance is from IrO_*x*_. Different conditions of 0.4 V/1.2 V and 0.4 V/1.0 V show similar results and are given in Fig. S2 of the supporting information. The charge values accumulated on the catalyst surface are acquired from the integral of the cathodic current curve to avoid the influence of OER current in the anodic process as shown in Fig. S3 of the supporting information. As a result, the total charge is 13.4 mC, 18.1 mC and 23.8 mC at the high potential of 1.0 V, 1.2 V and 1.4 V, respectively, which are almost proportional to the voltage.

### 4.2. Extraction and correction of the PSD demodulated spectrum

**Figure 5:**
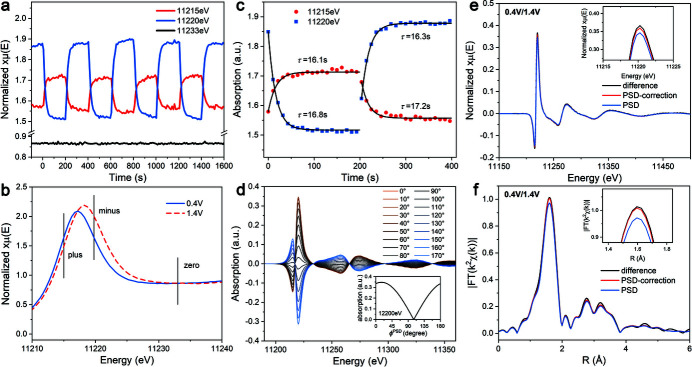
(*a*) Absorption coefficient changes with time at selected energy points. (*b*) Reconstructed original spectrum of two potentials and energy points selected. (*c*) The exponential fitting of the time response of the coordination coefficient with a square-wave excitation at different energy. (*d*) The PSD analysis of the phase lag induced by the relaxation of the system. (*e*, *f*) Comparison of the corrected and uncorrected PSD demodulated spectrum and the classical difference spectrum.

As described above, we continuously recorded XAFS during the electrochemical CA test which periodically changes the potential. Thus, we will obtain a full matrix of absorption coefficients μ(*E*, *t*) containing all the spectra changing with time. As seen in Fig. 5 ▸(*a*), the absorption coefficient shows good periodicity in two selected energy positions. We also find a point with no obvious vibration in the same scale plot, which proved that the vibration of the spectrum results from structural changes rather than other interference factors like the gathering of ions or the changing in liquid layer thickness, which will change similarly everywhere in the spectrum. We can also obtain two original spectra representing two different potentials via an average of the corresponding part of μ(*E*, *t*); the detailed method is shown in Fig. S4 of the supporting information and the results are shown in Fig. 5 ▸(*b*). It can be found that opposite phases at different energies in Fig. 5 ▸(*a*) represent opposite signs of the difference spectrum. Before performing equation (14), the kinetic parameter of the system should be obtained first, which was done with both fitting the relaxation curve and the PSD analysis. Fig. 5 ▸(*c*) shows the fitting results of the relaxation curve of the absorption coefficient, which fits the exponential function well. Details of the exponential fitting parameters are shown in Fig. S5 of the supporting information. The difference of the resulting time constants τ is not so significant for different energy and different re-dox process. Thus, it is reasonable to describe the system using equation (8). Although there are more elaborate models on the kinetic process of electrochemical systems, using the above model to correct the amplitude is already sufficient and further improvements should focus on experimental parameters. We also performed PSD analysis to obtain the kinetic parameter. As seen in Fig. 5 ▸(*d*), the amplitude of the demodulated spectrum varies in the form of a cos function, which fits equation (5). The amplitude takes its maximum value at 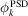 = 16.0°, which is the value of the phase lag. The corresponding time constant τ = 18.2 s is also obtained via equation (13). Since the fit to the relaxation curve strongly depends on the values of the first few points, the PSD results should be more reliable and used for further correction. Then, we calculated the amplitude attenuation via equation (13), which gave a result of 0.961, thus a 3.9% correction is needed. Figs. 5 ▸(*e*) and 5(*f*) show the corrected and non-corrected spectra compared with the difference spectrum obtained from the direct difference of the reconstructed original spectra. As a result, the difference between the corrected PSD demodulated spectrum and the difference spectrum is less than 2% in *E*-space and 1% in *R*-space. Considering the error in the measurement of the difference spectrum, the difference is acceptable, thus we can consider the corrected demodulated spectrum as the difference spectrum. In the following discussion, the amplitude-corrected PSD demodulated spectra are used for further analysis and we use the term ‘demodulated spectrum’ to refer to it.

### 4.3. Quantitative analysis of demodulated spectra

**Figure 6:**
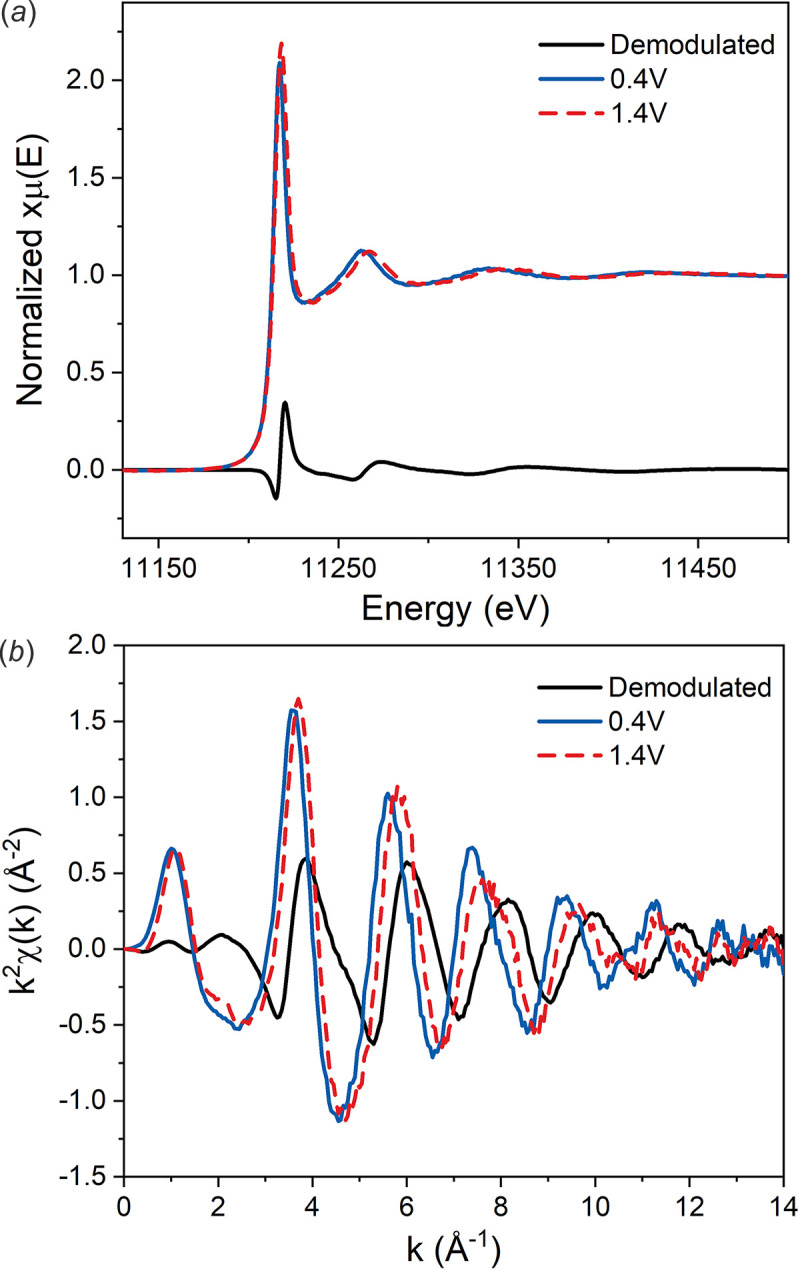
Comparison of the original spectra and the demodulated spectrum in *E*-space (*a*) and *k*-space (*b*).

The demodulated spectrum of IrO_*x*_ at two different potentials, 0.4 V and 1.4 V, and the corresponding reconstructed original spectra are obtained using the aforementioned approach. As shown in Fig. 6 ▸(*a*), the two spectra at different potentials are quite similar, indicating that the main phase of IrO_*x*_ has remained. The original spectrum at 1.4 V has a higher and blue-shifted white-line peak compared with 0.4 V, which is the typical feature of a higher oxidation state. As shown in Figs. 6 ▸(*a*) and 6(*b*), the demodulated spectrum has a smooth feature in both *E*-space and *k*-space even in the high-*k* range, implying a good S/N ratio. The most important characteristic of the demodulated spectrum is a ∼90° phase difference compared with the original spectrum. As simulated in former research (König *et al.*, 2012 ▸), the differential spectrum has no phase difference when coordination number and disorder changes, while 90° phase difference is typically observed if the bond length changes (Chu *et al.*, 2012 ▸). Thus, the change in bond length may be the most important factor.

**Figure 7:**
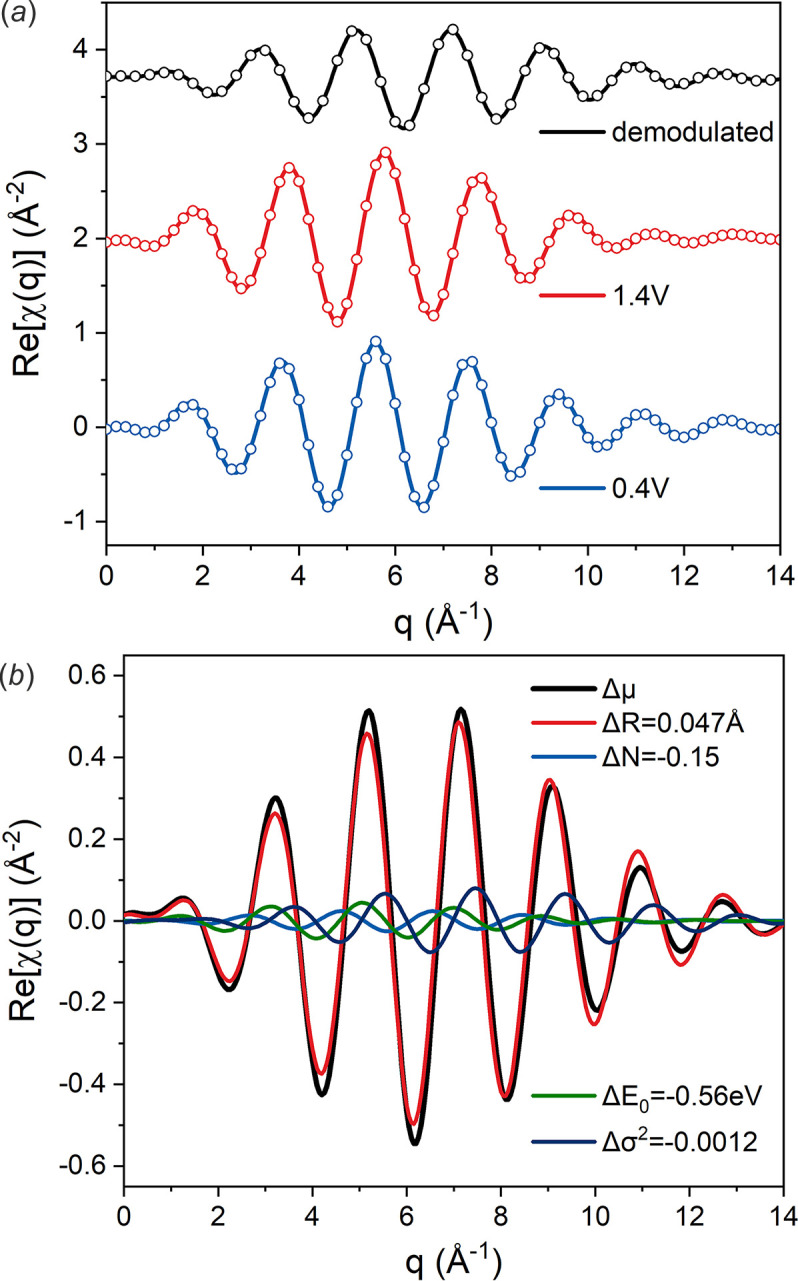
(*a*) Back Fourier transform (*R* range 1.2–1.95 Å) of the results of the combined fitting of the demodulated spectrum and original spectra. Circles: experimental data. Lines: fitted data. (*b*) The contributions of different changing parameters obtained from fitting.

Quantitative results are obtained via a combined fitting of the two original spectra and the demodulated spectrum. For the original spectra, one Ir–O path with four variables, *N*, *E*_0_, Δ*R* and σ^2^, were used. For fitting the demodulated spectrum, we used two Ir–O paths with the same variables as the corresponding original spectra, except one coordination number was set negative (Fig. S6 and Table S1 of the supporting information). Thus, the results equal the difference of the two spectra. A similar approach was used by König *et al.* (2012 ▸), who obtained the result of the static sample first and then use it to fit the difference. Considering the error of every single fitting, we choose to fit all of the spectrum in one fitting. As shown in Fig. 7 ▸(*a*), all the fitted curves have good consistency with the experimental data. The quantitative results are shown in Table 1 ▸. The difference in bond length is −0.047 Å, implying shrinking of the lattice at high potential. For a better comparison of the contribution of different parameters to the whole demodulated spectrum, we have simulated the differential spectrum where we let only one parameter change. As shown in Fig. 7 ▸(*b*), the most prominent factor is the bond length, while other fitting parameters exhibit a much smaller contribution, as in the aforementioned qualitative analysis.

**Figure 8:**
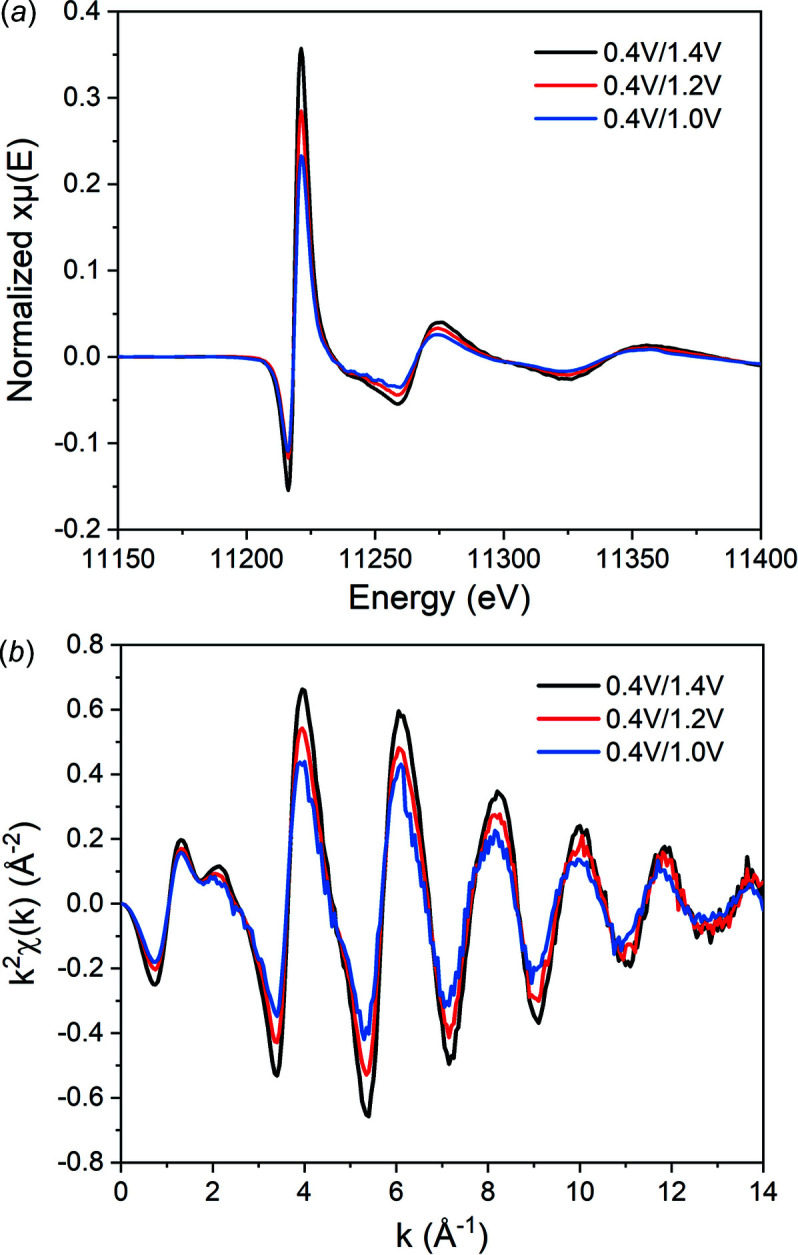
Comparison of the demodulated spectra of different modulation conditions in *E*-space (*a*) and *k*-space (*b*).

Three different oxidation potentials, 0.4 V/1.0 V, 1.4 V/1.2 V and 0.4 V/1.4 V, have been applied to confirm the findings. The demodulated spectra obtained using the PSD method have been corrected and compared with the traditional difference spectrum as seen in Fig. S7 of the supporting information. The good agreement between them again proves the correctness of this correction method. The results are shown in Figs. 8 ▸(*a*) and 8(*b*). The spectra remain smooth although the amplitude of the demodulated spectrum became smaller for low oxidation potential. These spectra are similar in both *E*-space and *k*-space, meaning that similar structural changes occur in the system. The amplitude of the demodulated spectrum increases almost linearly with the potential. For contrast, we tested normal *in situ* spectra with static potential and made their differential spectra by direct subtraction. As shown in Fig. S8 of the supporting information, it is obvious that without modulation and filter the spectra have poor quality, especially in the high-*k* range. Also, their change is not so regular compared with the spectra obtained from modulation. The above results demonstrate the advantages of the modulation approach. The main advantage is a better S/N ratio resulting from filtering. On the other hand, it is common that some irreversible change happens during the *in situ* test in OER (Cao, Shou *et al.*, 2021 ▸), while surface adsorption is a reversible process. Therefore, the modulated spectra show better regularity to potential since the result only comes from the reversible change of the absorption coefficient. In this regard, the modulation approach is more convincing in studies of the electrochemical adsorption process.

**Figure 9:**
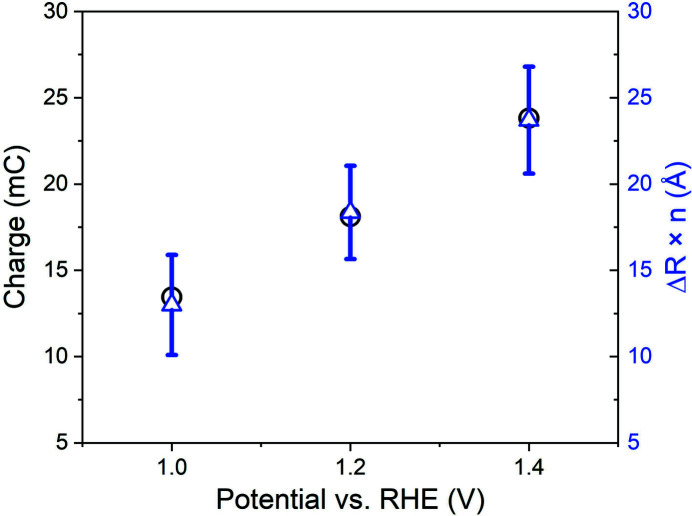
Comparison of the charge amount with the difference of Ir—O bond length for different modulation conditions.

Quantitative analysis is also performed for these spectra: the results are shown in Table 1 ▸ and the fitted curves are shown in Figs. S9–S11 of the supporting information. The difference in bond length improves with potential, while other parameters do not change significantly or within the error range. As shown in Fig. 9 ▸, a good proportional relationship is found between the charge amount and bond length. It is different from the potential induced phase transition which is a complete transformation only if the voltage exceeds the threshold. Considering the increase of oxidation state, we suppose that the shortening of the bond length at high potential is due to the detachment of protons (Kasian *et al.*, 2018 ▸).

## 5. Summary

In this article, we discussed the quantitative relationship between the PSD demodulated spectrum and difference spectrum. The amplitude attenuation is related to the modulation period and system relaxation time constant, which could be used to correct the amplitude. We propose a method for correcting the magnitude and give suggested experimental parameters. For good consistency, the condition *T*/τ > 20 should be satisfied. Then, we chose IrO_*x*_ as a model catalysis system and studied its reversible surface redox reaction. As a result, a high-quality demodulated spectrum has been obtained even in the high-*k* region and thus the differential EXAFS could be analyzed. We found that the change of bond length is almost proportional to the surface charge accumulation, which could offer a deeper understanding of the surface redox and adsorption–desorption process. Furthermore, modulating differential XAFS could be extended to wider applications in various electrochemical studies.

## 6. Resources

Information about the code for PSD analysis used here can be found at http://staff.ustc.edu.cn/~song2012/mes/mescode.htm.

## Supplementary Material

Table S1; Figures S1 to S11. DOI: 10.1107/S1600577522005616/ok5072sup1.pdf

## Figures and Tables

**Table 1:** EXAFS fitting results for different experiment parameters

		*R*-factor	Path	*N*	*E*_0_ (eV)	*R* (Å)	σ^2^ (10^−3^ Å^2^)
IrO_2_ standard		0.0073	Ir–O	6†	12.1 (12)	1.980 (8)	2.5 (5)
0.4 V/1.4 V	0.4 V		Ir–O	5.5 (3)	12.0 (6)	2.024 (4)	4.0 (6)
	1.4 V	0.0016	Ir–O	5.6 (2)	12.6 (6)	1.977 (5)	5.2 (6)
	Demodulated		Ir–O	−0.1 (3)	−0.6 (9)	0.047 (6)	−1.2 (8)
0.4 V/1.2 V	0.4 V		Ir–O	5.4 (2)	12.1 (5)	2.024 (4)	3.9 (5)
	1.2 V	0.0012	Ir–O	5.6 (1)	12.7 (5)	1.987 (4)	5.0 (6)
	Demodulated		Ir–O	−0.2 (3)	−0.6 (8)	0.037 (5)	−1.1 (8)
0.4 V/1.0 V	0.4 V		Ir–O	5.4 (3)	12.2 (6)	2.026 (5)	3.9 (6)
	1.0 V	0.0023	Ir–O	5.6 (2)	13.0 (5)	2.000 (4)	5.0 (5)
	Demodulated		Ir–O	−0.2 (4)	−0.8 (8)	0.026 (6)	−1.1 (8)

† Fixed variable.
