# Supplementary material for: Approach to electrochemical modulating differential extended X-ray absorption fine structure
Source: J Synchrotron Radiat. 2022 Jun 8;29(Pt 4):1065–73. doi: 10.1107/S1600577522005616 (PMC9255574; doi:10.1107/S1600577522005616)
Supplement: Supplementary file 1 [file s-29-01065-sup1.pdf]

JOURNAL OF SYNCHROTRON RADIATION

Volume 29 (2022)

Supporting information for article:

Approach to electrochemical modulating differential extended X-ray absorption fine structure

Wenjie Xu, Guikai Zhang, Hongwei Shou, Jia Zhou, Shuangming Chen, Shengqi Chu, Jing Zhang and Li Song

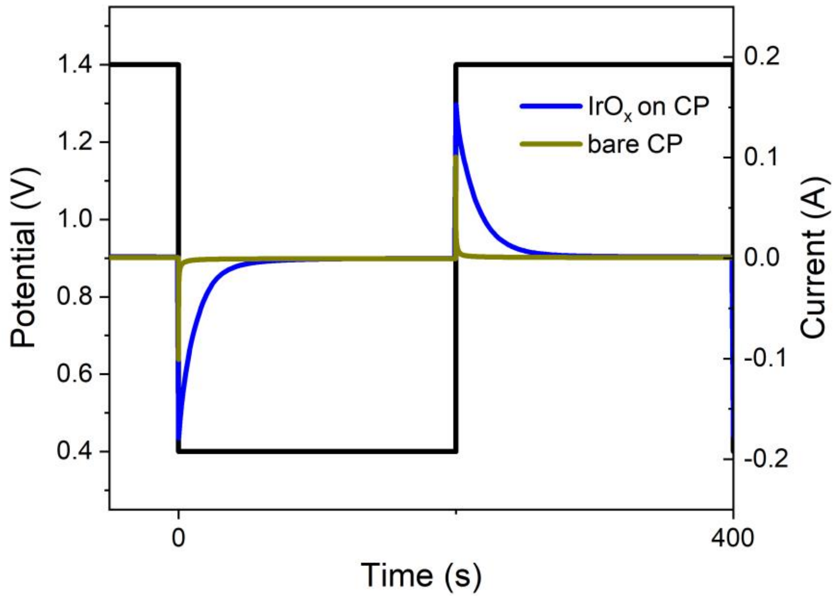

Figure S1 The CA curve of bare carbon.

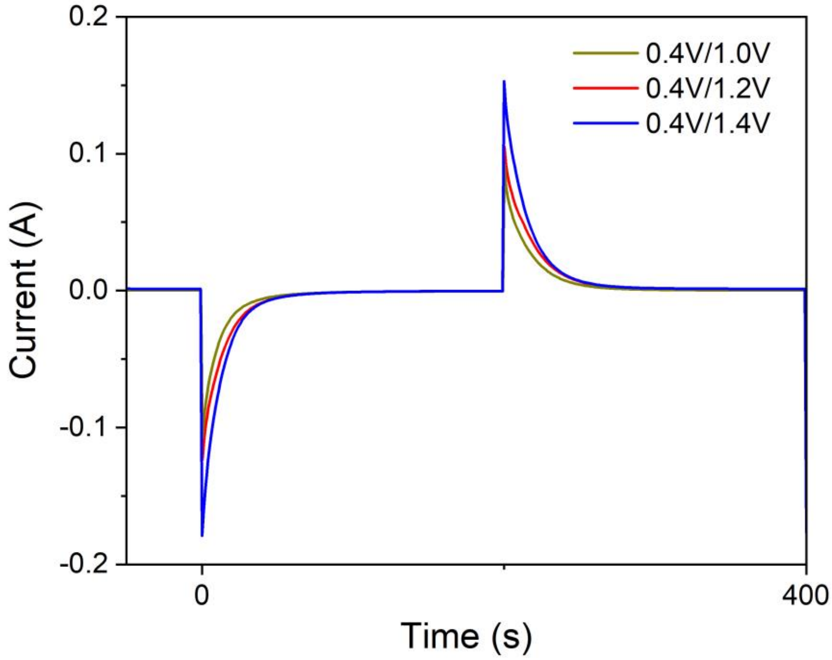

paper.

Figure S2 CA curves of different potential condition.

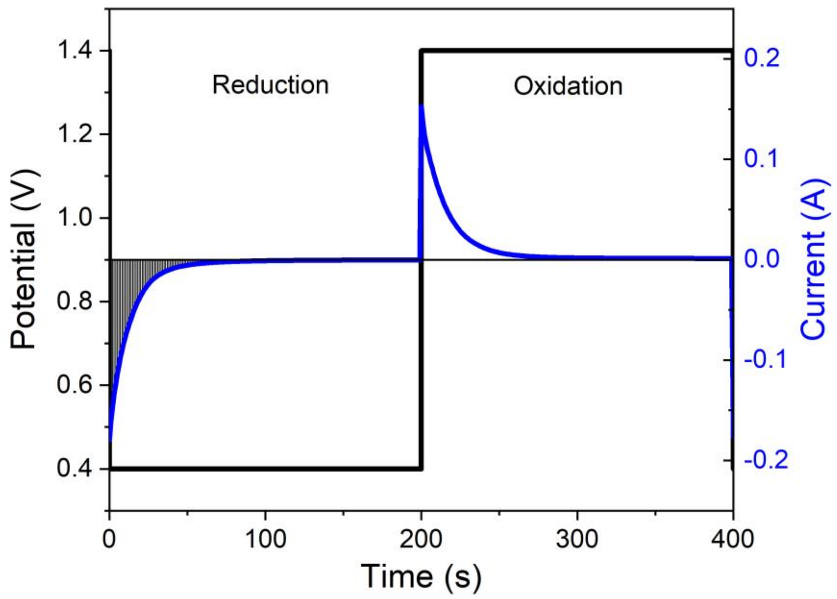

Figure S3 The method to calculate charge from CA curve. We calculated the amount of charge using the previous method (Nong *et al.*, 2020). Specifically, we integrated the current curve only in the reduction cycle in order to prevent the influence of OER current.

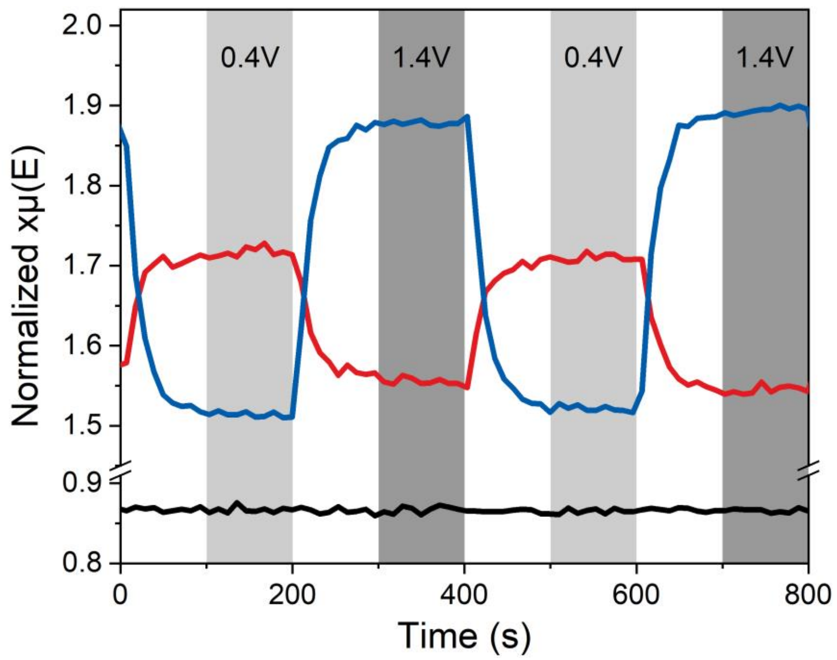

Figure S4 Range for reconstructing the original spectrum.

Nonlinear Curve Fit (ExpDec1) (2022/5/2 18:37:17)

Parameters

		Value	Standard Error	t-Value	Prob>|t|	Dependency
F	y0	1.5147	9.46784E-4	1599.83365	1.16468E-40	0.36108
	A1	0.33309	0.00316	105.52492	5.9278E-23	0.25538
	t1	16.83203	0.33319	50.51827	3.61121E-18	0.391
	k	0.05941	0.00118			
	tau	11.66707	0.23095			

Reduced Chi-sqr = 1.03090941212E-5
COD(R^2) = 0.99877740299082
Iterations Performed = 5
Total Iterations in Session = 5
Fit converged. Chi-Sqr tolerance value of 1E-9 was reached.
Standard Error was scaled with square root of reduced Chi-Sqr.
k, tau are derived parameter(s).

Fitted Curves Plot

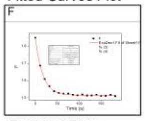

Residual Plots

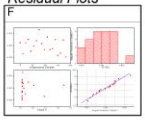

Nonlinear Curve Fit (ExpDec1) (2022/5/2 18:40:32)

Parameters

		Value	Standard Error	t-Value	Prob>|t|	Dependency
F	y0	1.87778	0.00127	1479.08968	1.60739E-42	0.33116
	A1	-0.253	0.00446	-56.74946	7.02115E-20	0.24561
	t1	16.25421	0.59676	27.23743	7.82314E-15	0.36782
	k	0.06152	0.00226			
	tau	11.26656	0.41364			

Reduced Chi-sqr = 2.04821932894E-5
COD(R^2) = 0.99552833691109
Iterations Performed = 5
Total Iterations in Session = 5
Fit converged. Chi-Sqr tolerance value of 1E-9 was reached.
Standard Error was scaled with square root of reduced Chi-Sqr.
k, tau are derived parameter(s).

Fitted Curves Plot

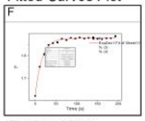

Residual Plots

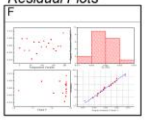

Nonlinear Curve Fit (ExpDec1) (2022/5/2 18:35:30)

Parameters

		Value	Standard Error	t-Value	Prob>|t|	Dependency
C	y0	1.71436	0.00189	908.73101	5.63326E-37	0.34734
	A1	-0.13341	0.00639	-20.89029	1.67714E-12	0.24705
	t1	16.14487	1.62212	9.95293	5.31673E-8	0.3759
	k	0.06194	0.00622			
	tau	11.19077	1.12437			

Reduced Chi-sqr = 4.18108367823E-5
COD(R^2) = 0.96963070259468
Iterations Performed = 8
Total Iterations in Session = 8
Fit converged. Chi-Sqr tolerance value of 1E-9 was reached.
Standard Error was scaled with square root of reduced Chi-Sqr.
k, tau are derived parameter(s).

Fitted Curves Plot

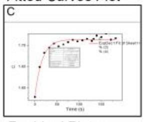

Residual Plots

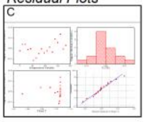

Nonlinear Curve Fit (ExpDec1) (2022/5/2 18:33:14)

Parameters

		Value	Standard Error	t-Value	Prob>|t|	Dependency
C	y0	1.55711	0.00181	858.21652	9.73727E-39	0.34842
	A1	0.11932	0.00625	19.09189	1.95657E-12	0.25646
	t1	17.16376	1.8607	9.22435	8.34488E-8	0.38705
	k	0.05826	0.00632			
	tau	11.89701	1.28974			

Reduced Chi-sqr = 4.07536308726E-5
COD(R^2) = 0.9619777130344
Iterations Performed = 8
Total Iterations in Session = 8
Fit converged. Chi-Sqr tolerance value of 1E-9 was reached.
Standard Error was scaled with square root of reduced Chi-Sqr.
k, tau are derived parameter(s).

Fitted Curves Plot

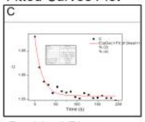

Residual Plots

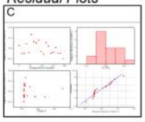

Figure S5 Fitting result of the relaxation curve of absorption coefficient to determine the time constant τ.

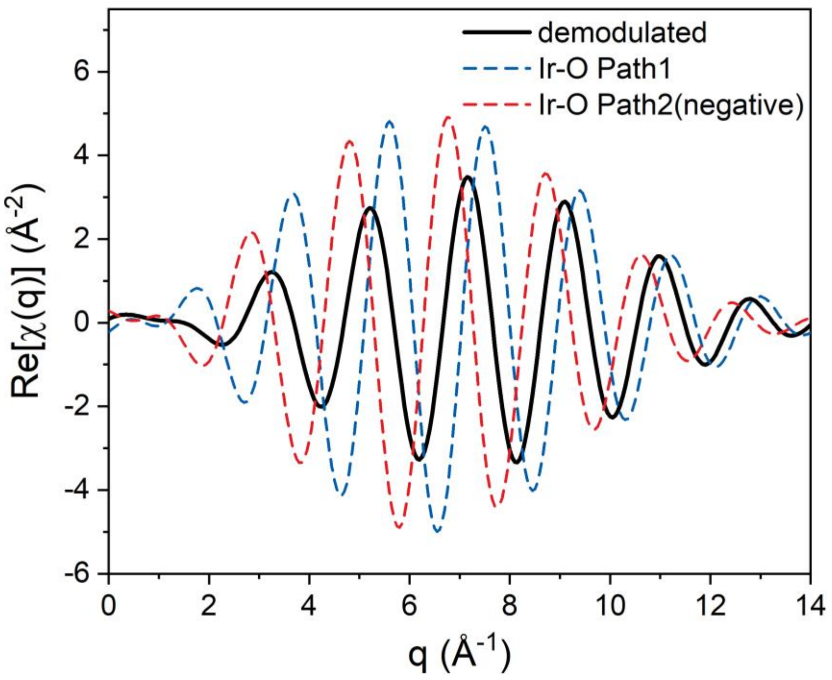

Figure S6 The paths used for fitting the demodulated spectrum.

Table S1 The fitting variables used for EXAFS fitting.

0.4V	Ir-O	N1	$\Delta E_0 1$	$\Delta R1$	$\sigma^2 1$
1.4V	Ir-O	N2	$\Delta E_0 2$	$\Delta R2$	$\sigma^2 2$
Demodulated	Ir-O	N1	$\Delta E_0 1$	$\Delta R1$	$\sigma^2 1$
	Ir-O	-N2	$\Delta E_0 2$	$\Delta R2$	$\sigma^2 2$

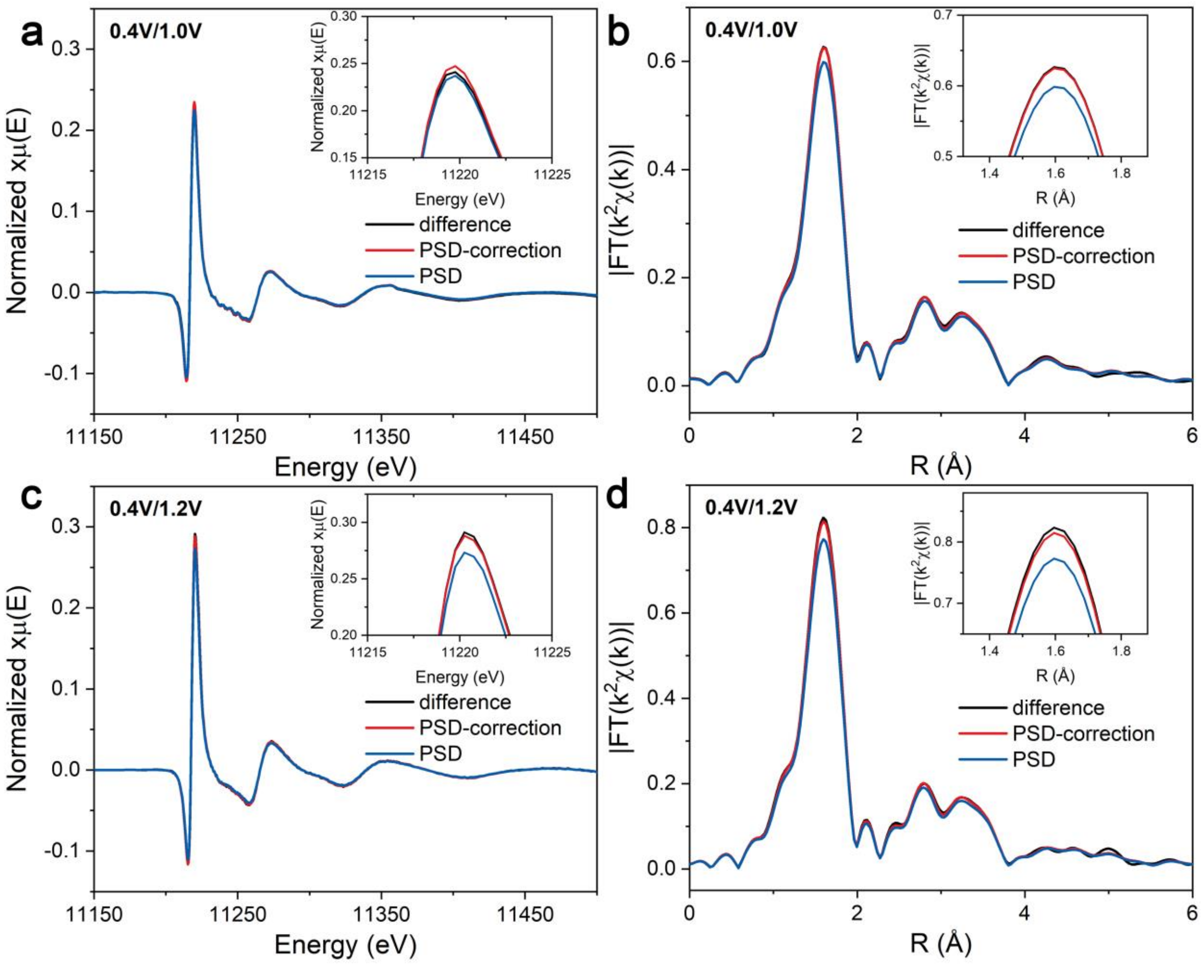

Figure S7 Correction results at other experiment conditions.

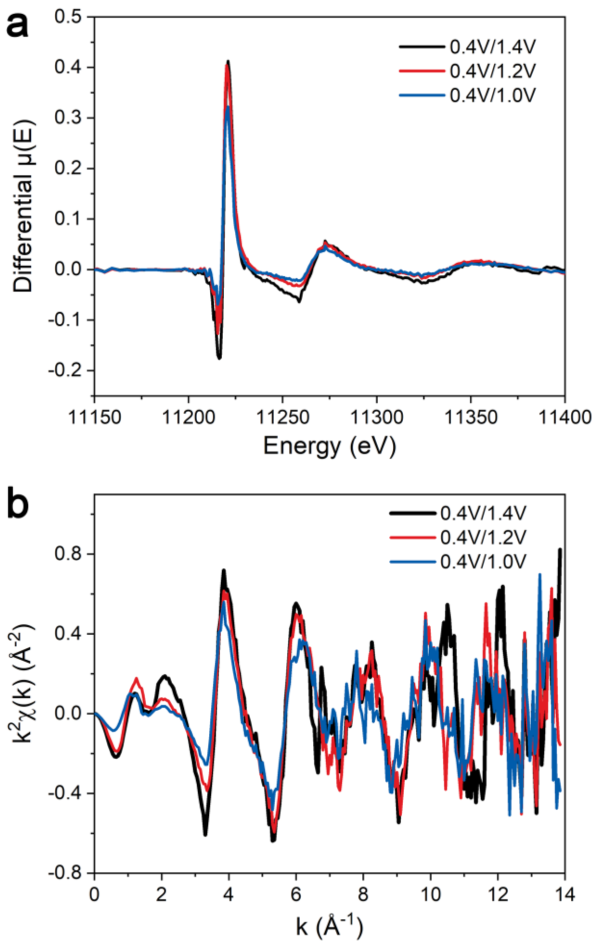

Figure S8 Difference spectra obtained from direct minus of normally tested in-situ XAFS where a static potential is applied.

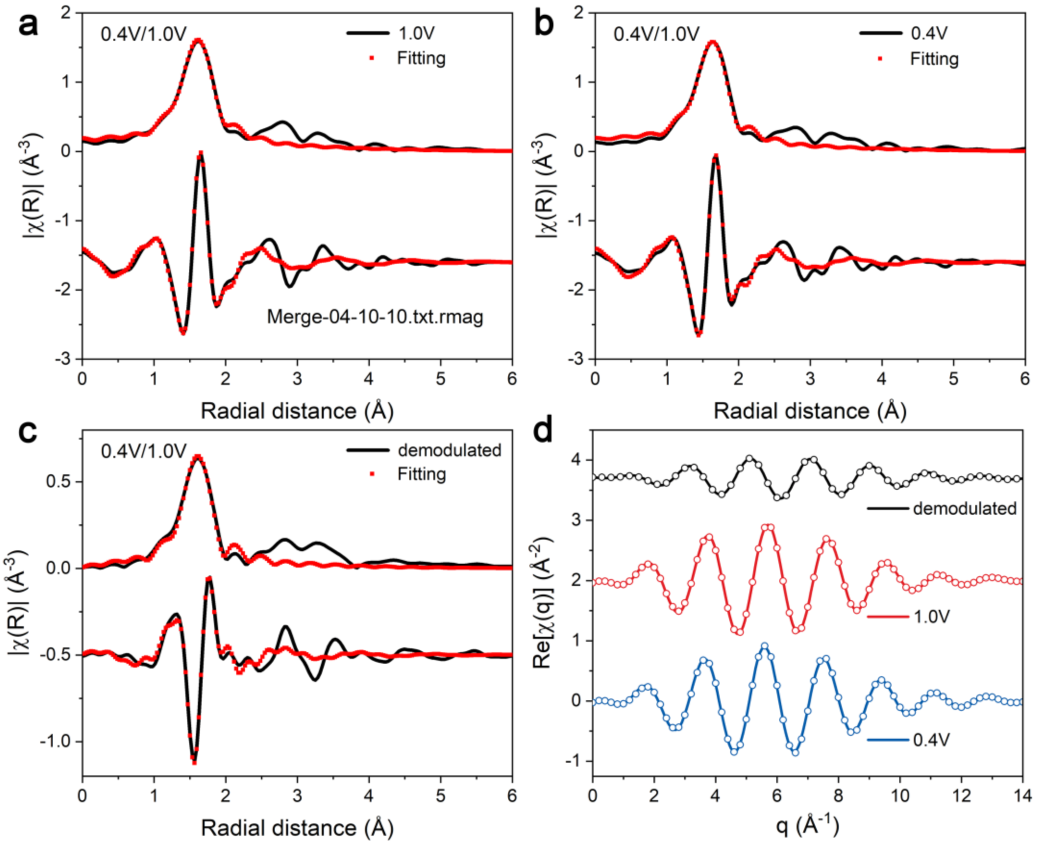

Figure S9 Fitted curves of modulating potential 0.4V/1.0V.

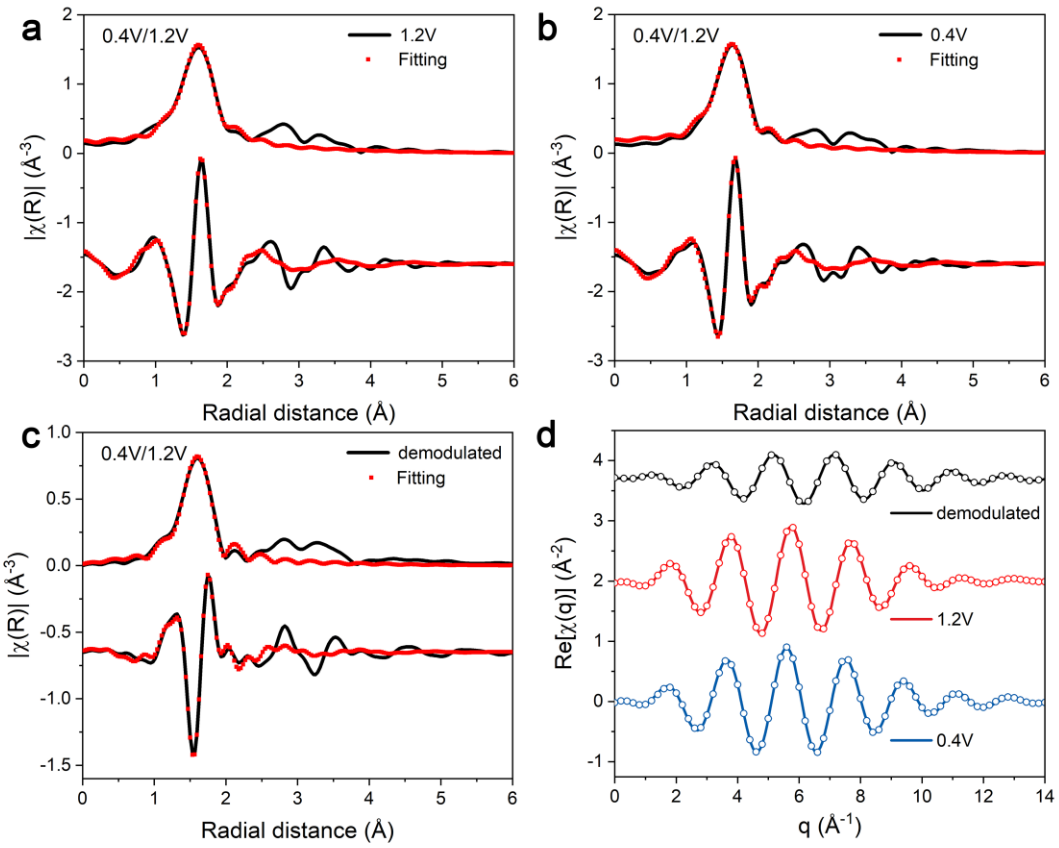

Figure S10 Fitted curves of modulating potential 0.4V/1.2V.

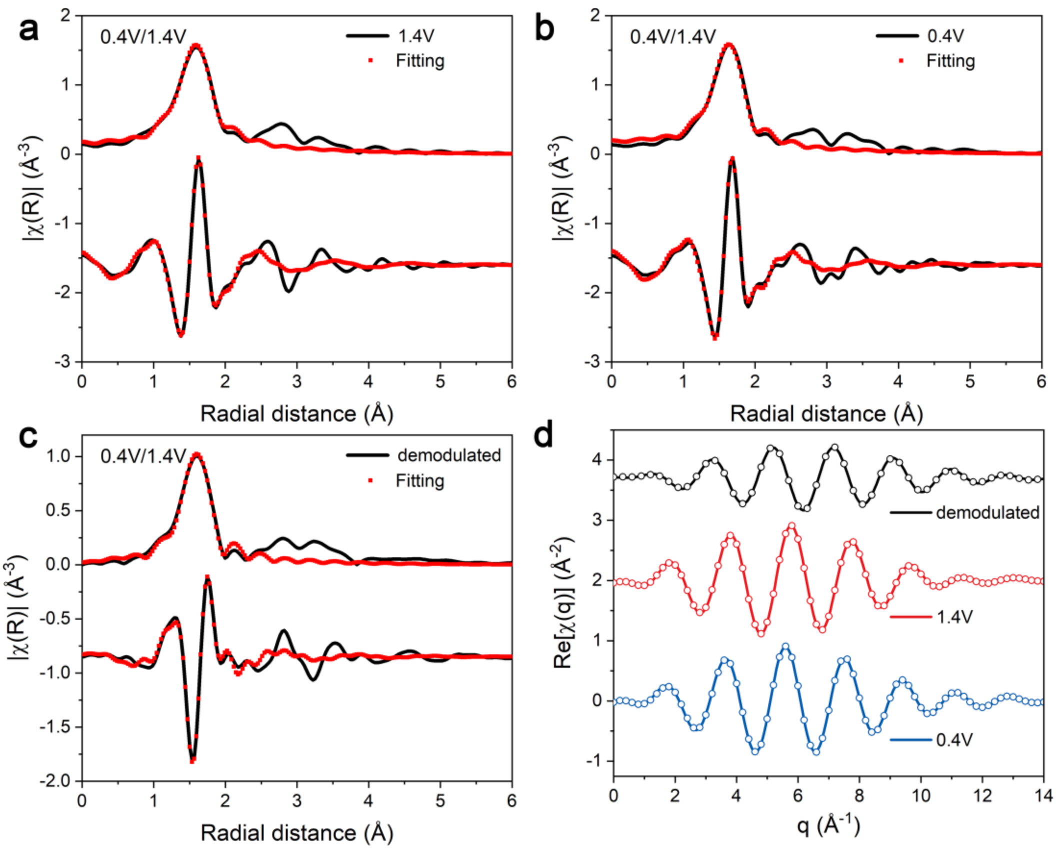

Figure S11 Fitted curves of modulating potential 0.4V/1.4V.

Reference

Nong, H. N., Falling, L. J., Bergmann, A., Klingenhof, M., Tran, H. P., Spori, C., Mom, R., Timoshenko, J., Zichittella, G., Knop-Gericke, A., Piccinin, S., Perez-Ramirez, J., Cuenya, B. R., Schlogl, R., Strasser, P., Teschner, D. & Jones, T. E. (2020). *Nature,* **587**, 408-413.
